# Supplementary material for: The Phaseolus vulgaris Receptor-Like Kinase PvFER1 and the Small Peptides PvRALF1 and PvRALF6 Regulate Nodule Number as a Function of Nitrate Availability
Source: Int J Mol Sci. 2023 Mar 9;24(6):5230. doi: 10.3390/ijms24065230 (PMC10049175; doi:10.3390/ijms24065230)
Supplement: Supplementary file 1 [file ijms-24-05230-s001.zip › ijms-2178213-supplementary.pdf]

Supplementary Material

The *Phaseolus vulgaris* receptor-like kinase PvFER1 and the small peptides PvRALF1 and PvRALF6 regulate nodule number as a function of nitrate availability

Jorge Solís-Miranda[1], Marco A. Juárez-Verdayes[1,2], Noreide Nava[1], Paul Rosas[1], Alfonso Leija-Salas[3], Luis Cárdenas[1] and Carmen Quinto[1*]

[1] Departamento de Biología Molecular de Plantas, Instituto de Biotecnología, Universidad Nacional Autónoma de México, Avenida Universidad 2001, Colonia Chamilpa, Cuernavaca, Morelos 62210, México jorge.solis@ibt.unam.mx, noreide.nava@ibt.unam.mx, paul.rosas@ibt.unam.mx, luis.cardenas@ibt.unam.mx
[2] Present address: Departamento de Docencia, Universidad Autónoma Agraria Antonio Narro, Saltillo, Coahuila 25315, México; relf125@hotmail.com
[3] Centro de Ciencias Genómicas, Universidad Nacional Autónoma de México, Avenida Universidad 2001, Colonia Chamilpa, Cuernavaca, Morelos 62210, México. leija@ccg.unam.mx
* Corresponding author: carmen.quinto@ibt.unam.mx

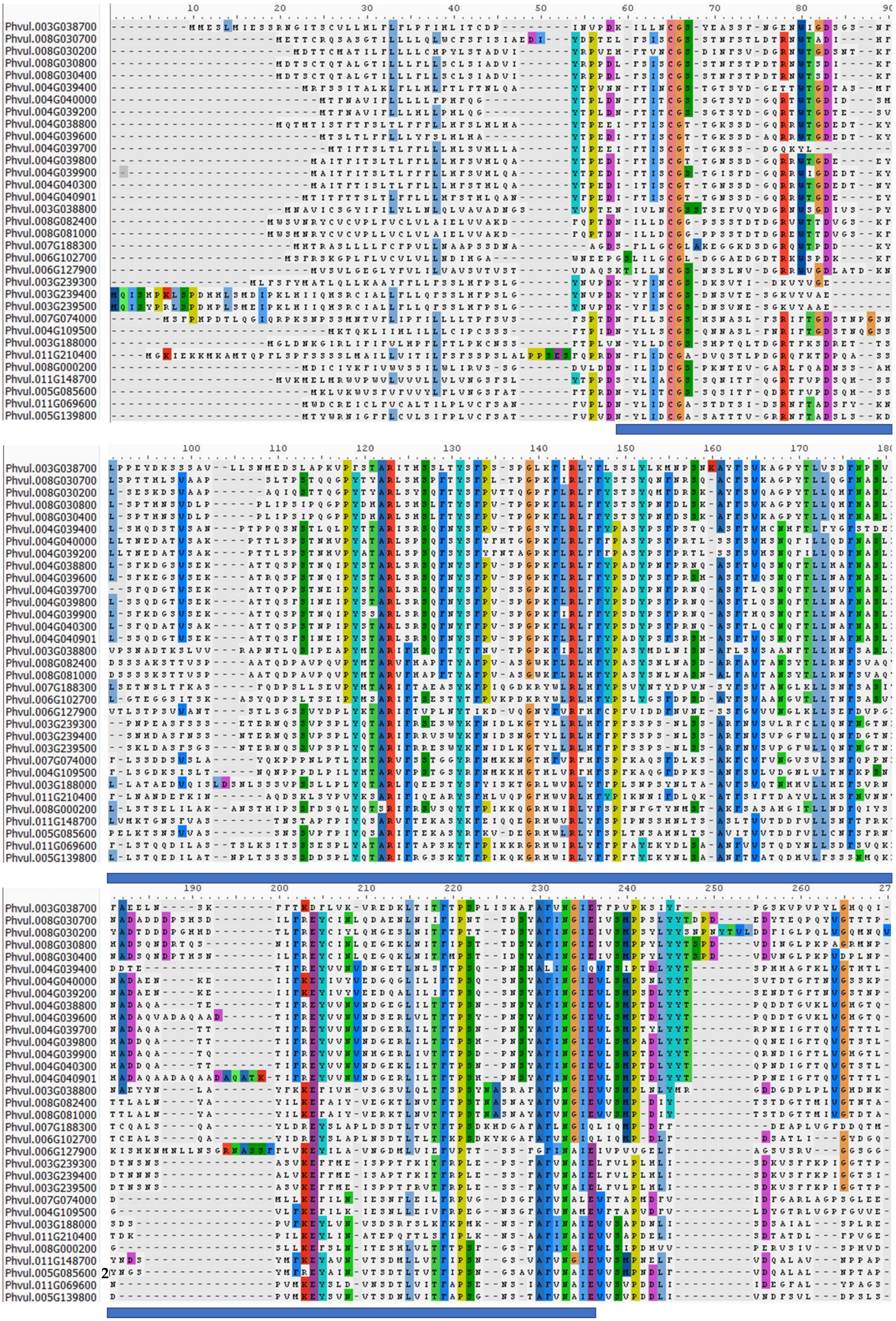
Phvul.003G038700
Phvul.008G030700
Phvul.008G030200
Phvul.008G030800
Phvul.008G030400
Phvul.004G039400
Phvul.004G040000
Phvul.004G039200
Phvul.004G038800
Phvul.004G039600
Phvul.004G039700
Phvul.004G039800
Phvul.004G039900
Phvul.004G040300
Phvul.004G040901
Phvul.003G038800
Phvul.008G082400
Phvul.008G081000
Phvul.007G188300
Phvul.006G102700
Phvul.006G127900
Phvul.003G239300
Phvul.003G239400
Phvul.003G239500
Phvul.007G074000
Phvul.004G109500
Phvul.003G188000
Phvul.011G210400
Phvul.008G000200
Phvul.011G148700
Phvul.005G085600
Phvul.011G069600
Phvul.005G139800

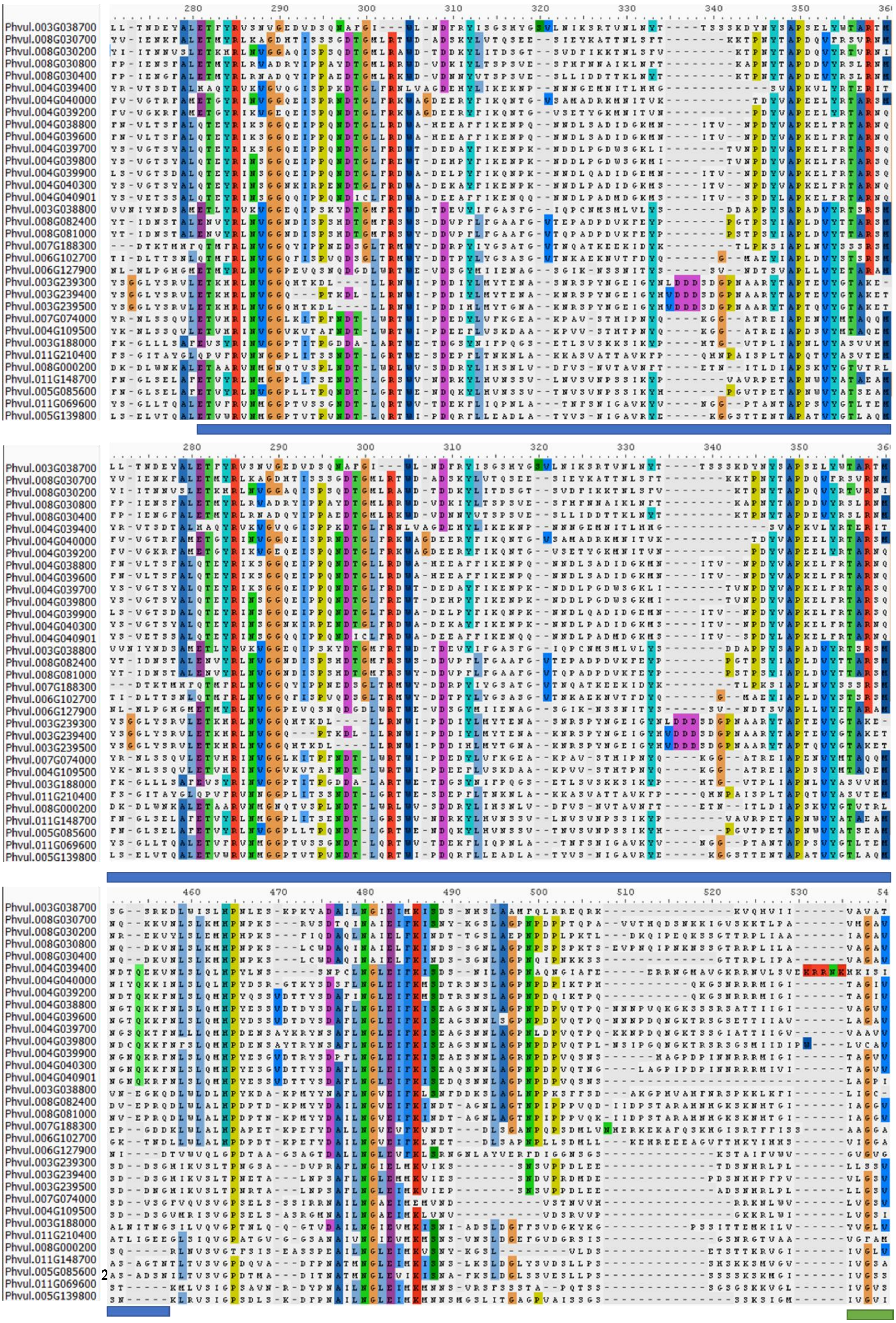
Phvul.003G038700
Phvul.008G030700
Phvul.008G030200
Phvul.008G030800
Phvul.008G030400
Phvul.004G039400
Phvul.004G040000
Phvul.004G039200
Phvul.004G038800
Phvul.004G039600
Phvul.004G039700
Phvul.004G039800
Phvul.004G039900
Phvul.004G040300
Phvul.004G040901
Phvul.003G038800
Phvul.008G082400
Phvul.008G081000
Phvul.007G188300
Phvul.006G102700
Phvul.006G127900
Phvul.003G239300
Phvul.003G239400
Phvul.003G239500
Phvul.007G074000
Phvul.004G109500
Phvul.003G188000
Phvul.011G210400
Phvul.008G000200
Phvul.011G148700
Phvul.005G085600
Phvul.011G069600
Phvul.005G139800

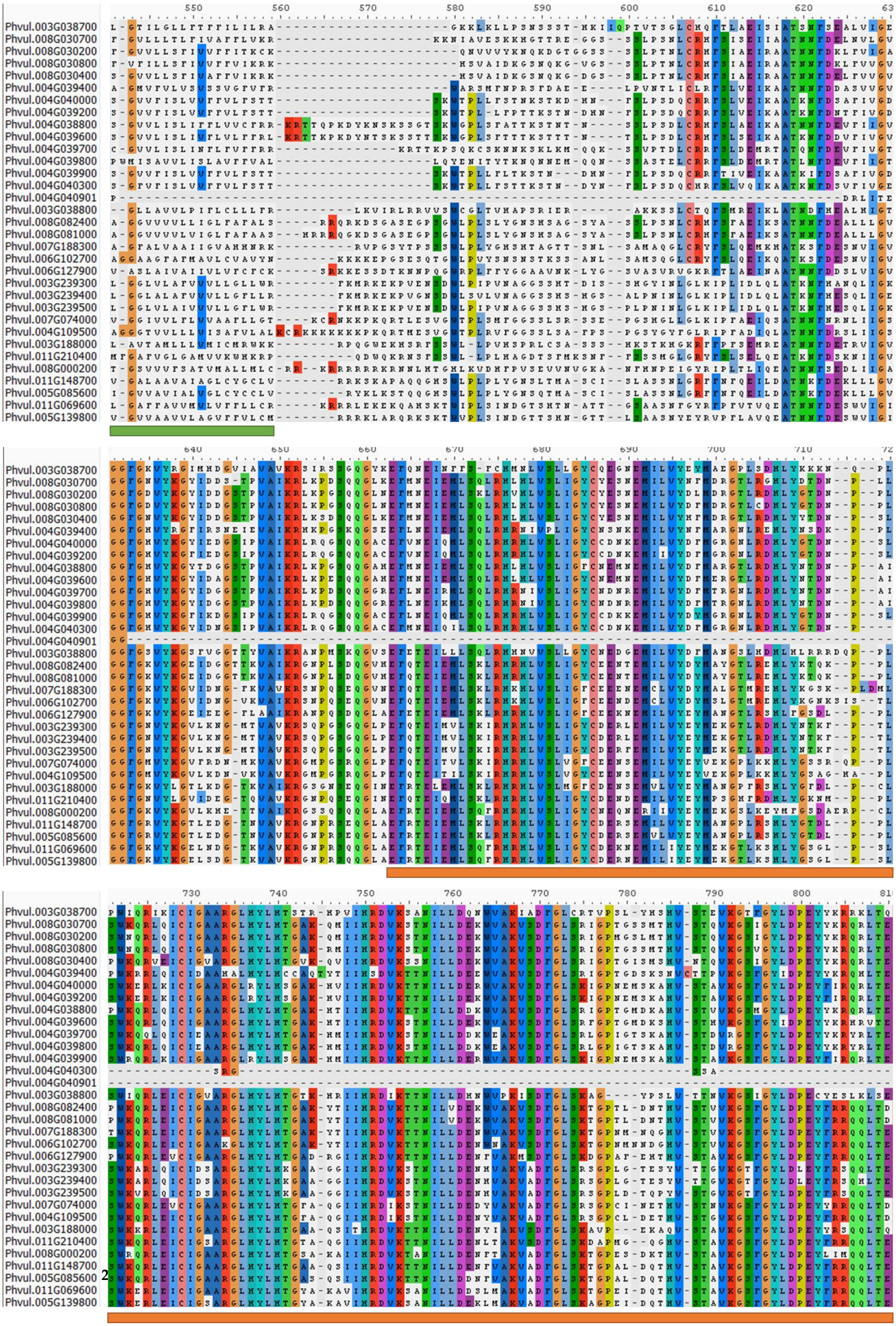
550
560
570
580
590
600
610
620
Phvul.003G038700
Phvul.008G030700
Phvul.008G030200
Phvul.008G030800
Phvul.008G030400
Phvul.004G039400
Phvul.004G040000
Phvul.004G039200
Phvul.004G038800
Phvul.004G039600
Phvul.004G039700
Phvul.004G039800
Phvul.004G039900
Phvul.004G040300
Phvul.004G040901
Phvul.003G038800
Phvul.008G082400
Phvul.008G081000
Phvul.007G188300
Phvul.006G102700
Phvul.006G127900
Phvul.003G239300
Phvul.003G239400
Phvul.003G239500
Phvul.007G074000
Phvul.004G109500
Phvul.003G188000
Phvul.011G210400
Phvul.008G000200
Phvul.011G148700
Phvul.005G085600
Phvul.011G069600
Phvul.005G139800
640
650
660
670
680
690
700
710
730
740
750
760
770
780
790
800

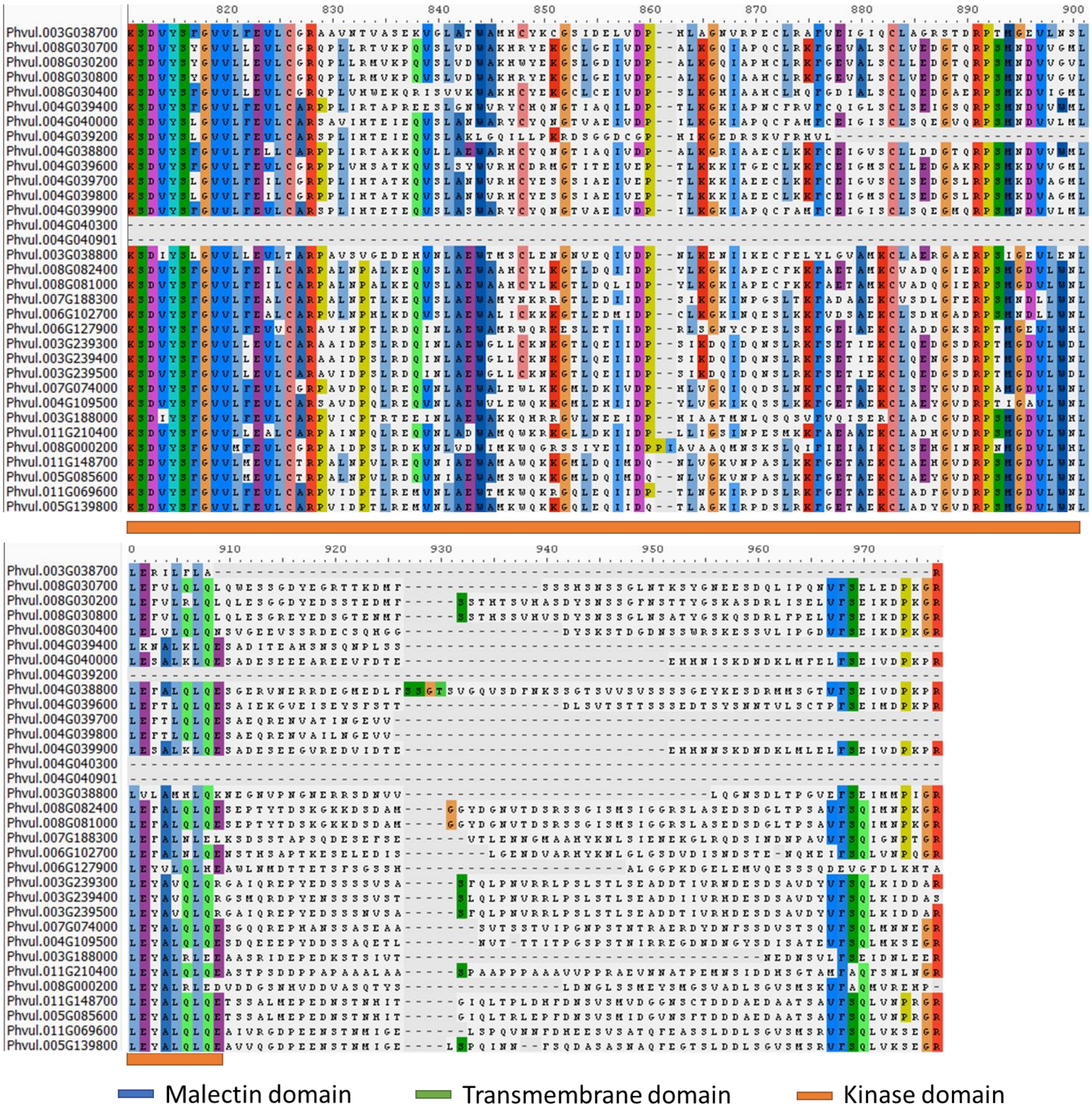

Fig. S1. Sequence alignment of *P. vulgaris* CrRLK1L protein sequences. Aligned amino acid sequences of the 33 CrRLK1Ls from *P. vulgaris*. The majority consensus amino acids are highlighted. Common domains of the CrRLK1L subfamily are indicated by colored lines: blue, malectine domain; green, transmembrane domain; orange, kinase domain.

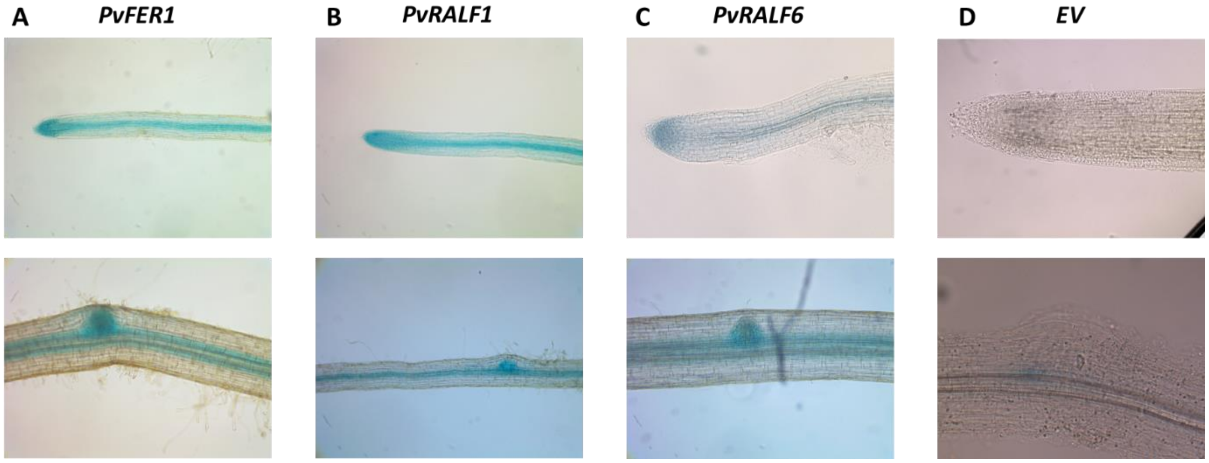

Fig. S2. Spatiotemporal activity of *PvFER1*, *PvRALF1*, and *PvRALF6* promoters and empty vector (EV) in transgenic *P. vulgaris* roots. Bright-field images of transgenic *P. vulgaris* roots expressing pro*PvFER1*::GUS (A), pro*PvRALF1*::GUS (B), and pro*PvRALF6*::GUS (C). The activity of each of the promoters was determined by GUS staining in the roots (upper panels) and lateral root primordia (lower panels). Bright-field images of transgenic P. vulgaris roots expressing the pBGWFS7.0 empty vector lacking a promoter to control the expression of the GUS reporter (D).

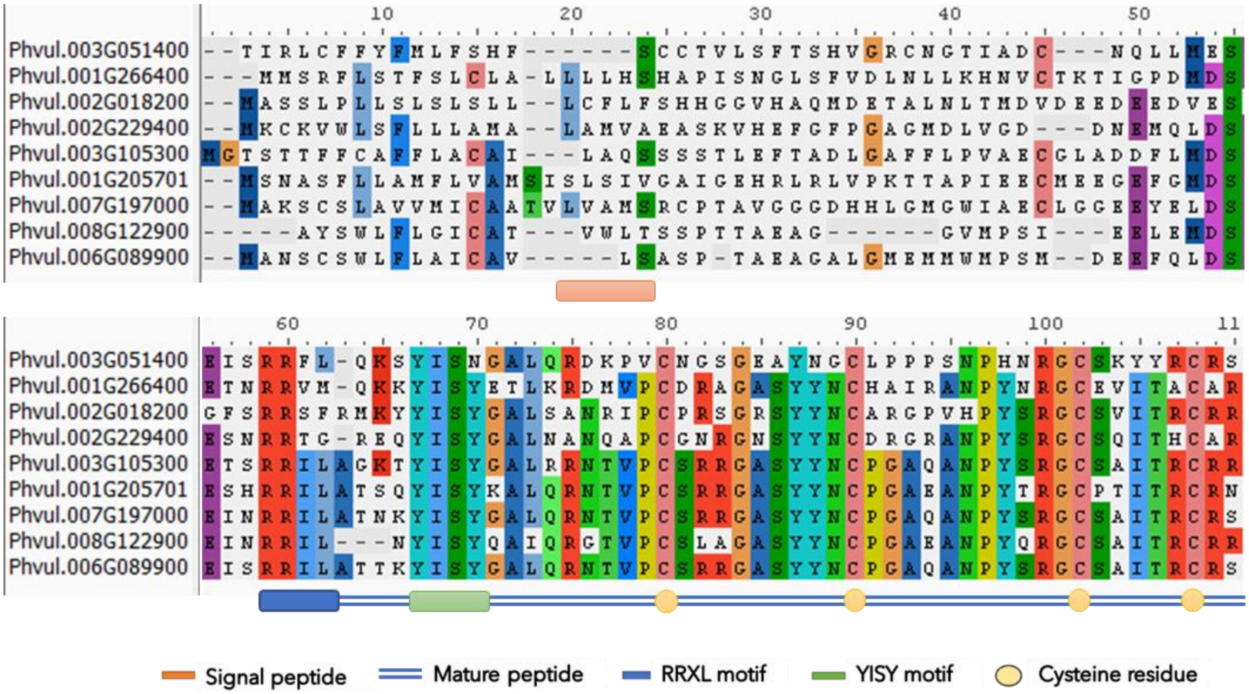

Fig. S3. Sequence alignment of *P. vulgaris* RALF protein sequences. Aligned amino acid sequences of the nine RALFs (B) from *P. vulgaris*. The majority consensus amino acids are highlighted. Common features of the RALF family are indicated by the different colors: orange, signal peptide; magenta line, mature peptide sequence; blue, RRXL motif; green, YISI motif; yellow circle, cysteine residue.

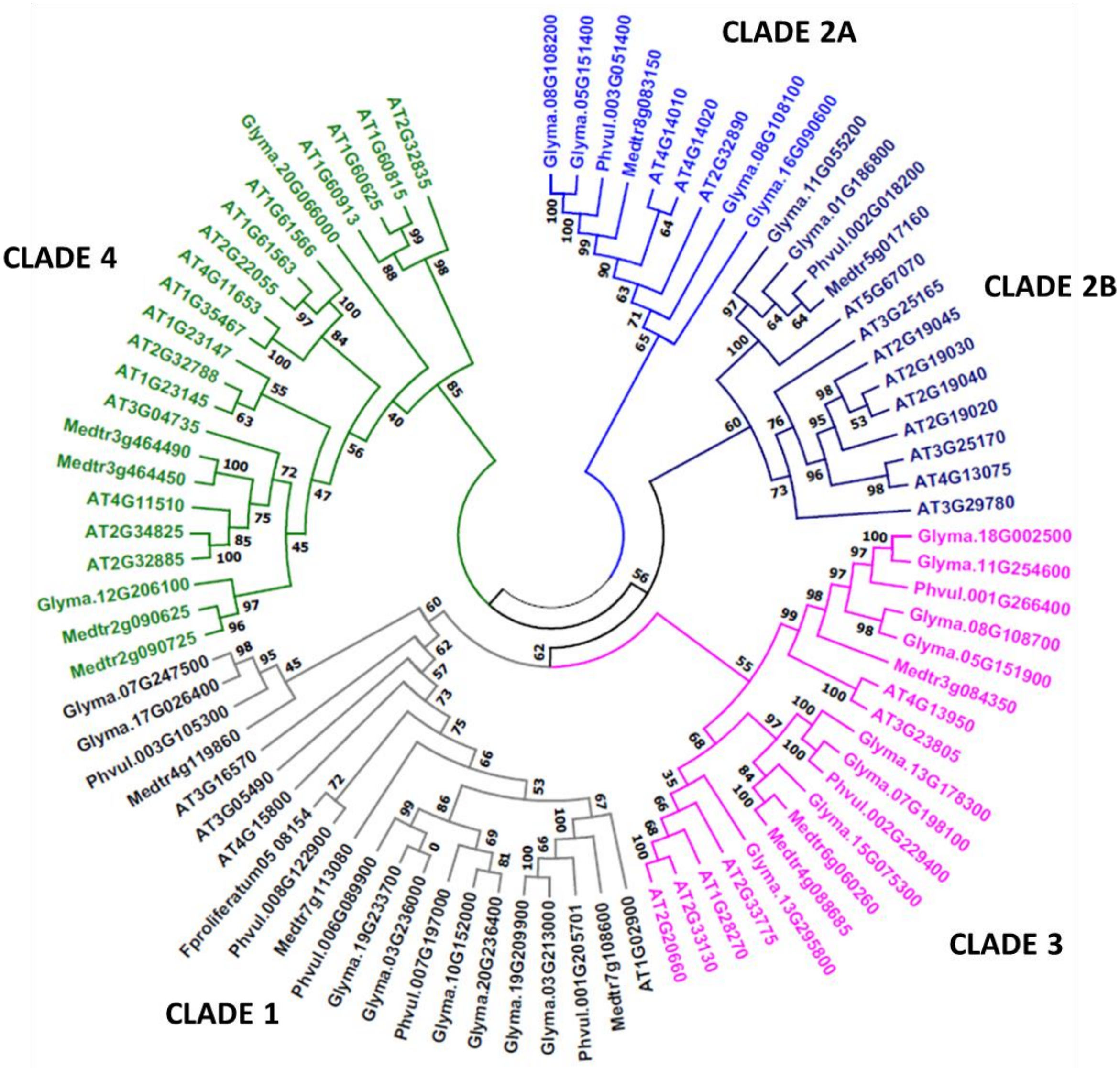

Gene ID	Name	Clade
Phvul.007G197000	PvRALF1	1
Phvul.008G122900	PvRALF2	1
Phvul.006G089900	PvRALF3	1
Phvul.001G205701	PvRALF5	1
Phvul.003G105300	PvRALF9	1
Phvul.002G018200	PvRALF7	2B
Phvul.003G051400	PvRALF8	2A
Phvul.002G229400	PvRALF4	3
Phvul.001G266400	PvRALF6	3

Fig. S4. Evolutionary relationships among RALFs. RALF proteins from *A. thaliana, P. vulgaris, G. max, M. truncatula,* and *Fusarium proliferatum* form four major clades, as indicated by different colors. P. vulgaris RALF genes ID, names, and clades are summarized in the bottom table.

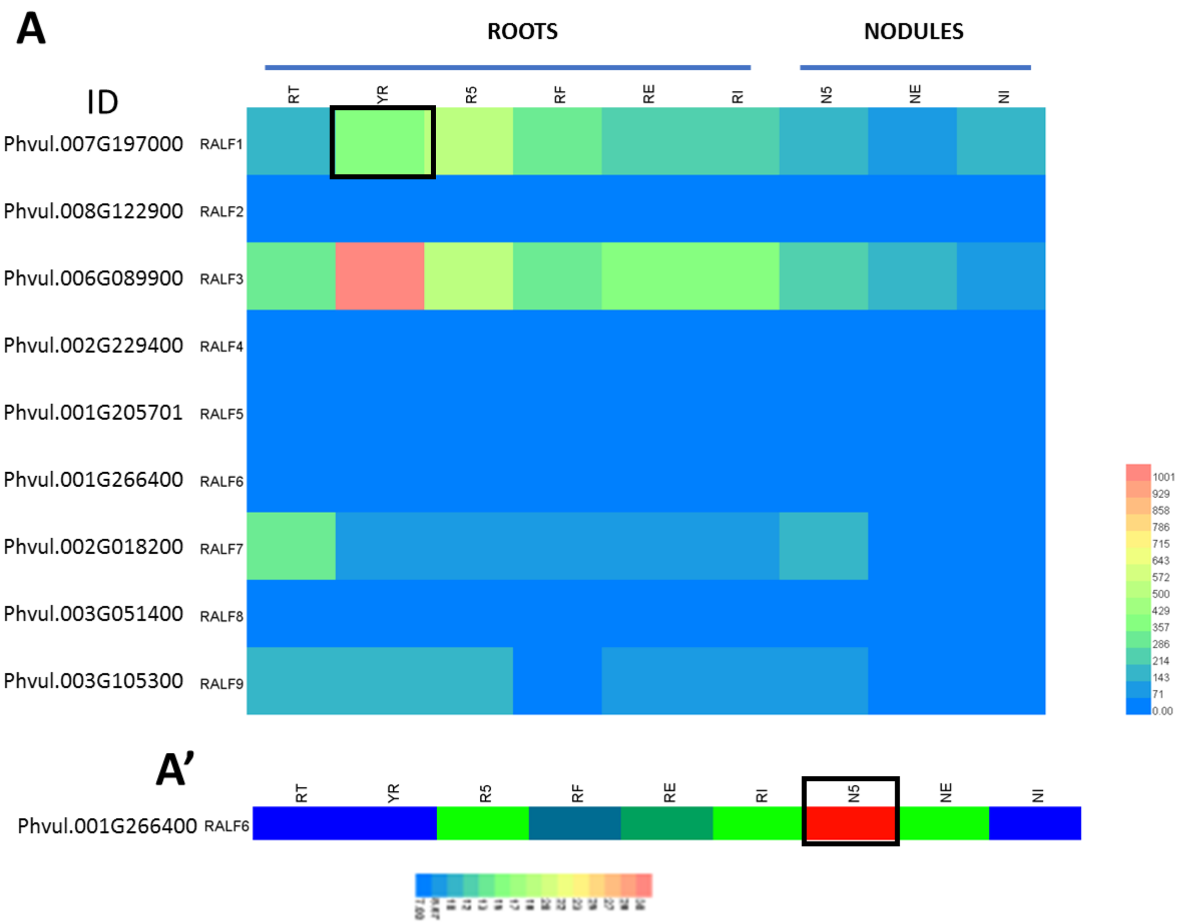

Fig. S5. Expression profiles of *PvRALF* genes in *P. vulgaris* roots and nodules. A) Heat map depicting *PvRALF* expression profiles in *P. vulgaris*. A') Expression levels of *PvRALF6* only in *P. vulgaris* roots and nodules. The transcriptome data were extracted from O'Rourke *et al*. (2014). RPKM values are represented as key color codes next to each heat map. RT, root apices collected from fertilized plants; TR, whole roots; R5, 5 dpi denodulated roots; RF, fertilized whole roots collected at the same time as RE and RI; RE, 21 dpi denodulated roots inoculated with WT rhizobia; RI, 21 dpi denodulated roots inoculated with fix- rhizobia; N5, 5 dpi nodules, NE, 21 dpi fixing + nodules; NI, 21 dpi fixing - nodules.

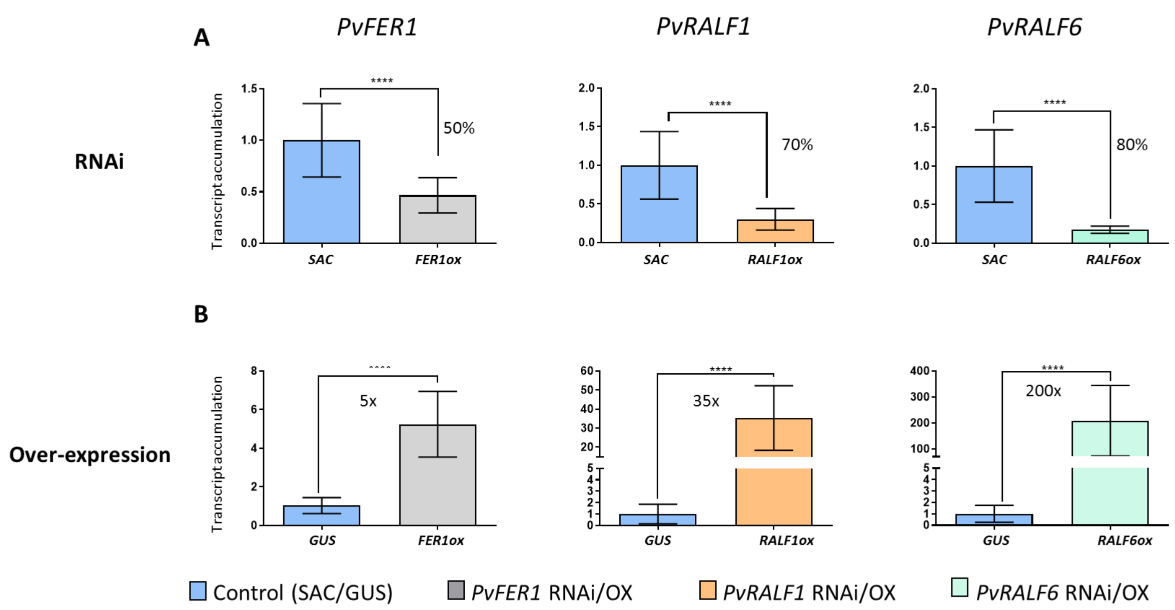

Fig. S6. Expression levels of *PvFER1*, *PvRALF1*, and *PvRALF6* in silenced and overexpressed transgenic roots. Relative expression levels of *PvFER1*, *PvRALF1*, and *PvRALF6* in silenced (A) and overexpressed (B) transgenic roots. *PvFER1i*, *PvRALF1i*, and *PvRALF6i* are transgenic silenced roots (A); *PvFER1ox*, *PvRALF1ox*, and *PvRALF6ox* are overexpressing transgenic roots (B); and SAC and GUS are controls for the silenced and overexpressed plants, respectively. Numbers in A and B indicate the percentage of silencing or fold increase in expression compared with the control roots. Bars represent the data means ± SD ($n = 9$). A non-parametric Mann-Whitney test was used to assess significant differences. ****$P < 0.001$. The transcript accumulation of the *IDE* gene was used as a reference.

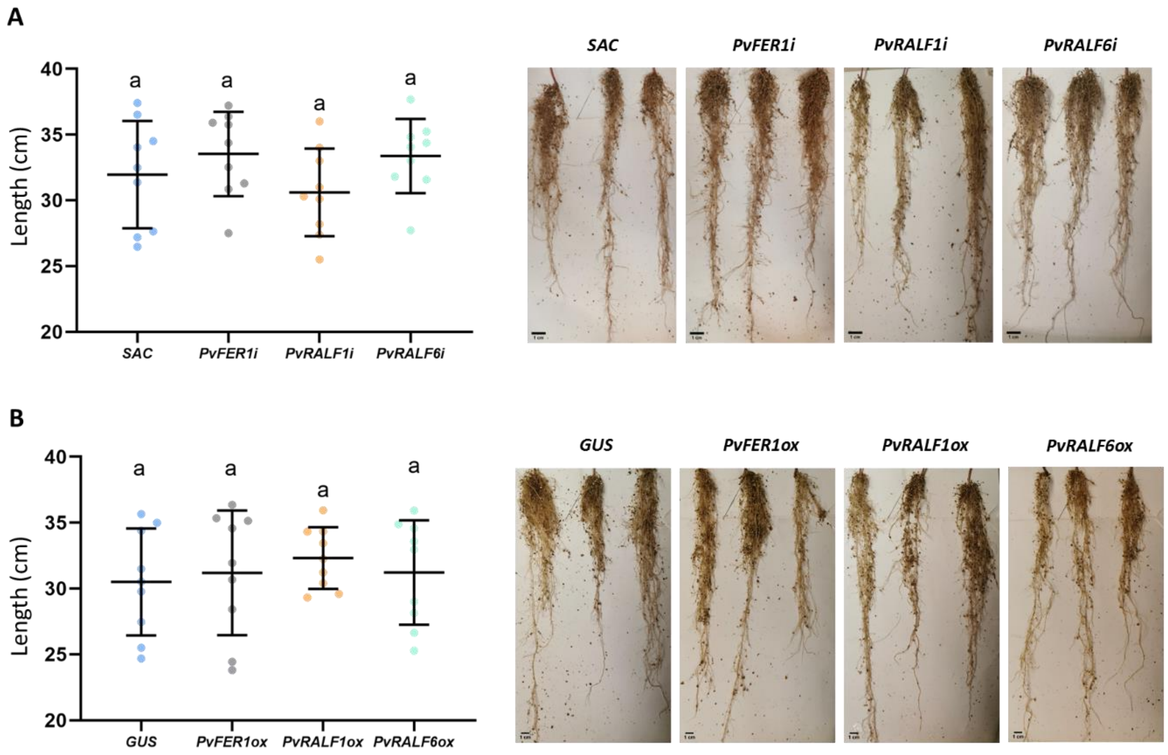

Fig. S7. Root lengths of *PvFER1*-, *PvRALF-1*-, and *PvRALF6*-silenced and -overexpressing transgenic roots at 21 dpi. A, B) Lengths of *PvFER1*-, *PvRALF1*-, and *PvRALF6*-silenced (A) and -overexpressing (B) roots and their respective controls (SAC or GUS). Lines represent the mean ± SD. Data from three independent experiments are plotted as dots ($n = 9$). A non-parametric Kruskal-Wallis test followed by Dunn's multiple comparisons was used to assess significant differences, with a P value < 0.05 represented by different letters at the top of the bar.

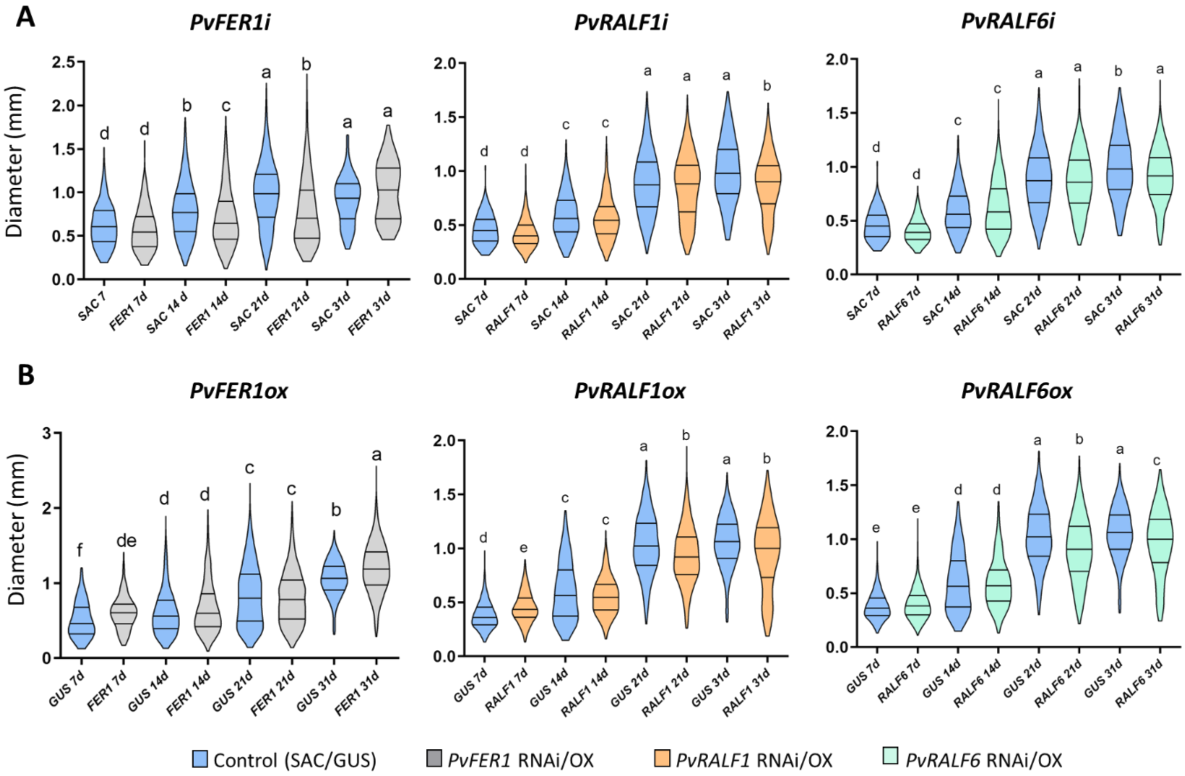

Fig. S8. Nodule diameters in *PvFER1*-, *PvRALF1*-, and *PvRALF6*-silenced and -overexpressing roots at 0 mM nitrate. Analysis of the nodule diameter of *PvFER1*- (gray violin plots), *PvRALF1*- (orange violin plots), and *PvRALF6*-silenced (green violin plots) (upper panels) and -overexpressing (lower panels) transgenic roots, as well as the control roots (blue violin plots) in 0 mM nitrate at 7, 14, 21, and 31 dpi. Violin plots represent the distribution of all data ($n \geq 100$), the horizontal line within the box represents the median and the upper, and lower lines delimit the interquartile range. A non-parametric Kruskal-Wallis test followed by Dunn's multiple comparisons was used to assess significant differences.

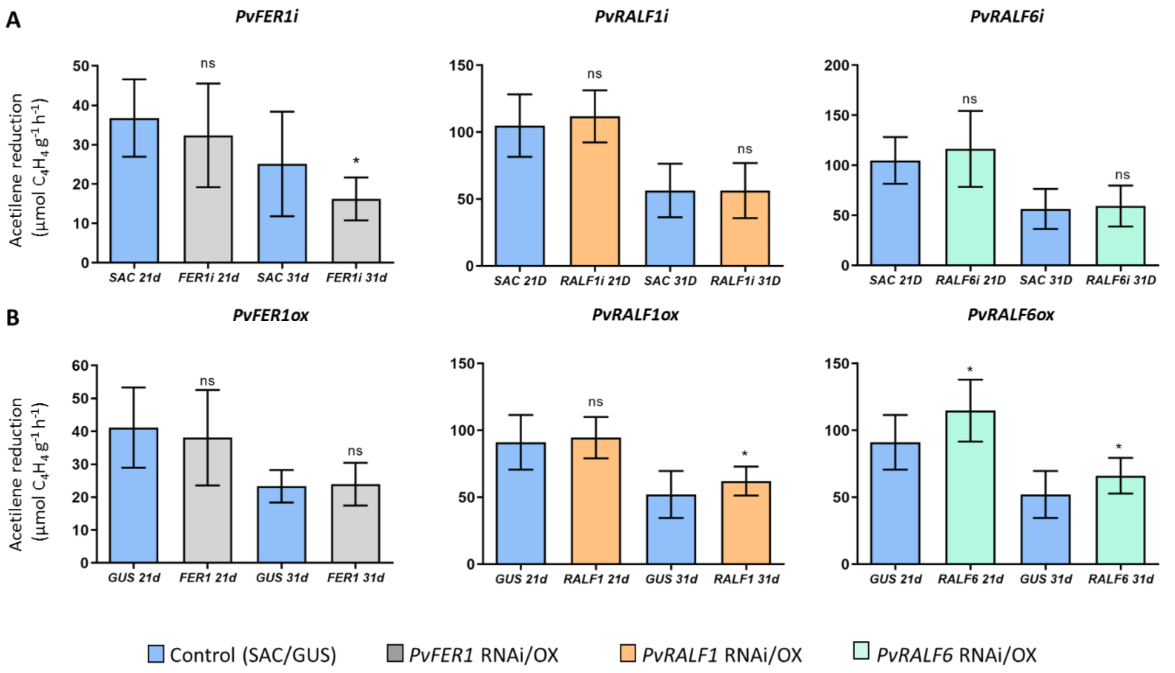

Fig. S9. Measurement of acetylene reduction of *PvFER1-*, *PvRALF1-*, and *PvRALF6*-silenced and -overexpressing transgenic roots in 0 mM nitrate. Acetylene-reducing activity in *PvFER1-* (gray bars), *PvRALF1-* (orange bars), and *PvRALF6*-silenced (green bars) (A) and -overexpressing (B) transgenic roots at 21 and 31 dpi in 0 mM nitrate. Blue bars represent control roots (SAC or GUS). Bars represent the data means ± SD ($n = 15$). A non-parametric Mann-Whitney test was used to assess significant differences. ns = non-significant, $^{*}P < 0.05$.

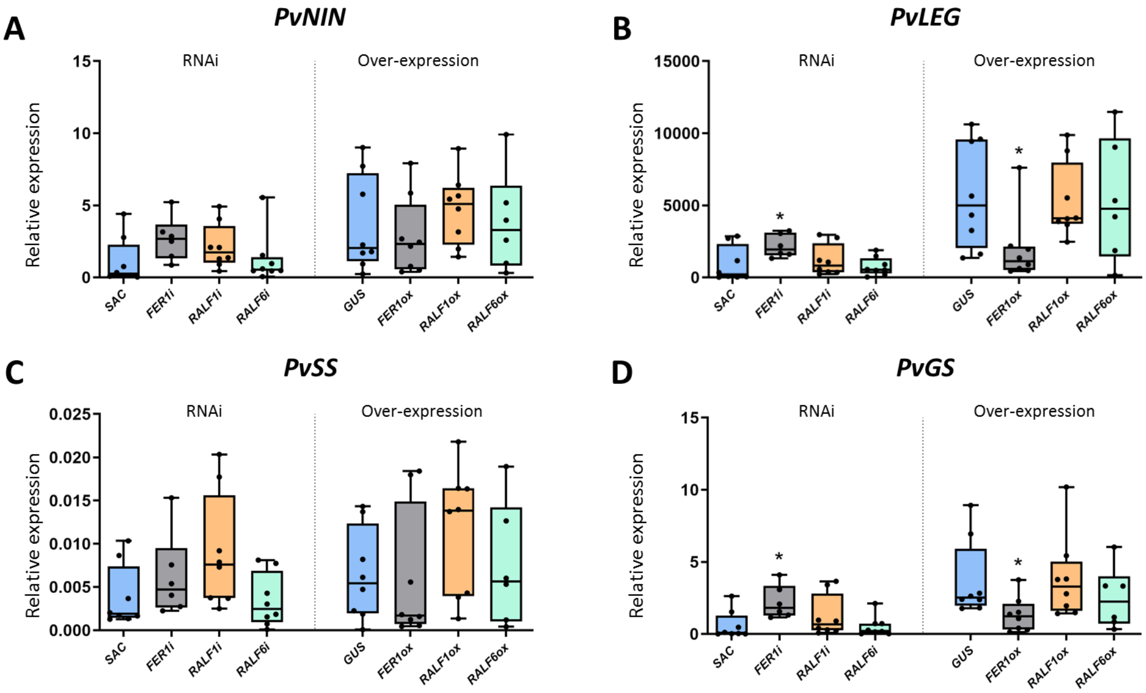

Fig. S10. Analysis of the accumulation of transcripts of marker genes for the organogenesis and metabolism of nodules in transgenic roots in 0 mM nitrate. Relative expression levels of *PvNIN* (A), *PvLEG* (B), *PvSS* (C), and *PvGS* (D) genes in transgenic roots at 21 dpi under low-nitrate conditions. *PvFER1i*, *PvRALF1i*, and *PvRALF6i* are silenced transgenic roots, and *PvFER1ox*, *PvRALF1ox*, and *PvRALF6ox* are overexpressed transgenic roots. The upper and lower edges of the boxes delimit the first to third quartiles, the horizontal line within the box represents the median, and the whiskers indicate the smallest and largest outlier in the data set. Points represent all data ($n \geq 8$). A non-parametric Mann-Whitney test was used to assess significant differences. *$P < 0.05$. The transcript accumulation of the *IDE* gene was used as a reference.

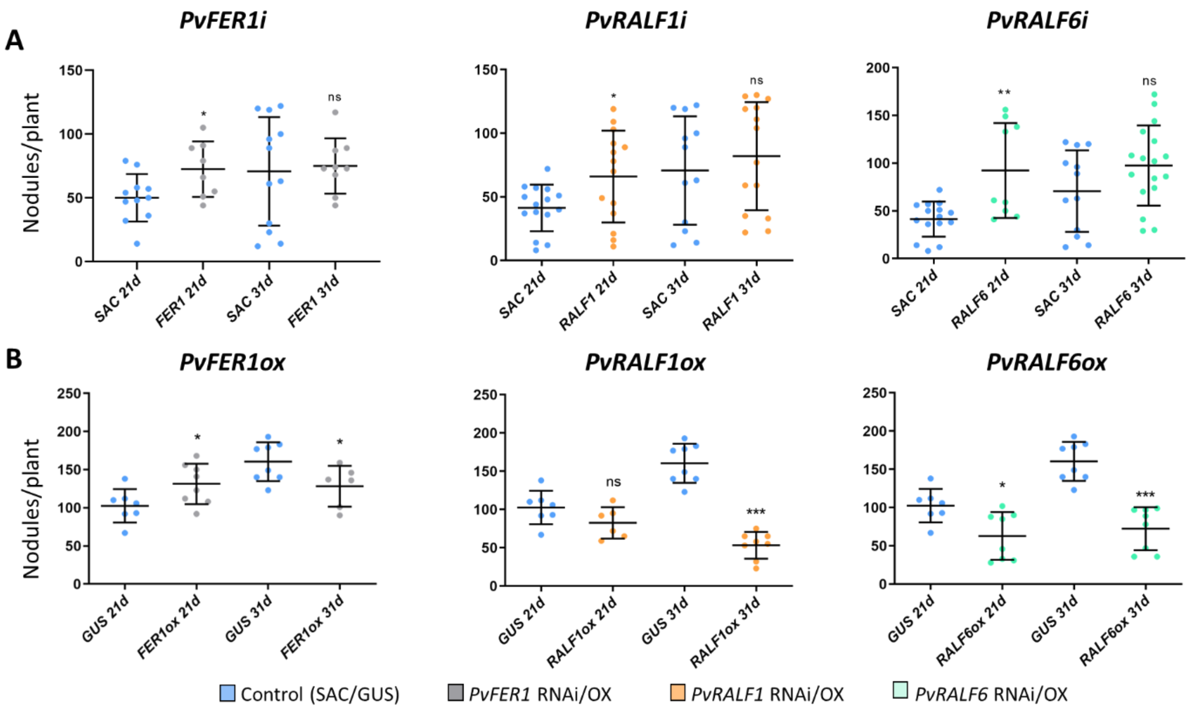

Fig. S11. Analysis of the number of nodules in *P. vulgaris* roots with silenced or overexpressed *PvFER1*, *PvRALF1*, and *PvRALF6* genes under 2 mM nitrate. Effect of the RNAi silencing (A) and overexpression (B) of *PvFER1* (gray dots), *PvRALF1* (orange dots), and *PvRALF6* (green dots) on nodule number at 21 and 31 dpi under 2 mM nitrate. The control is represented as blue dots. The lines represent the means ± SD. Data from three independent experiments are represented as points ($n \geq 9$). A non-parametric Kruskal-Wallis test was used to assess significant differences. ns = non-significant, *$P < 0.05$, **$P < 0.01$, ***$P < 0.001$.

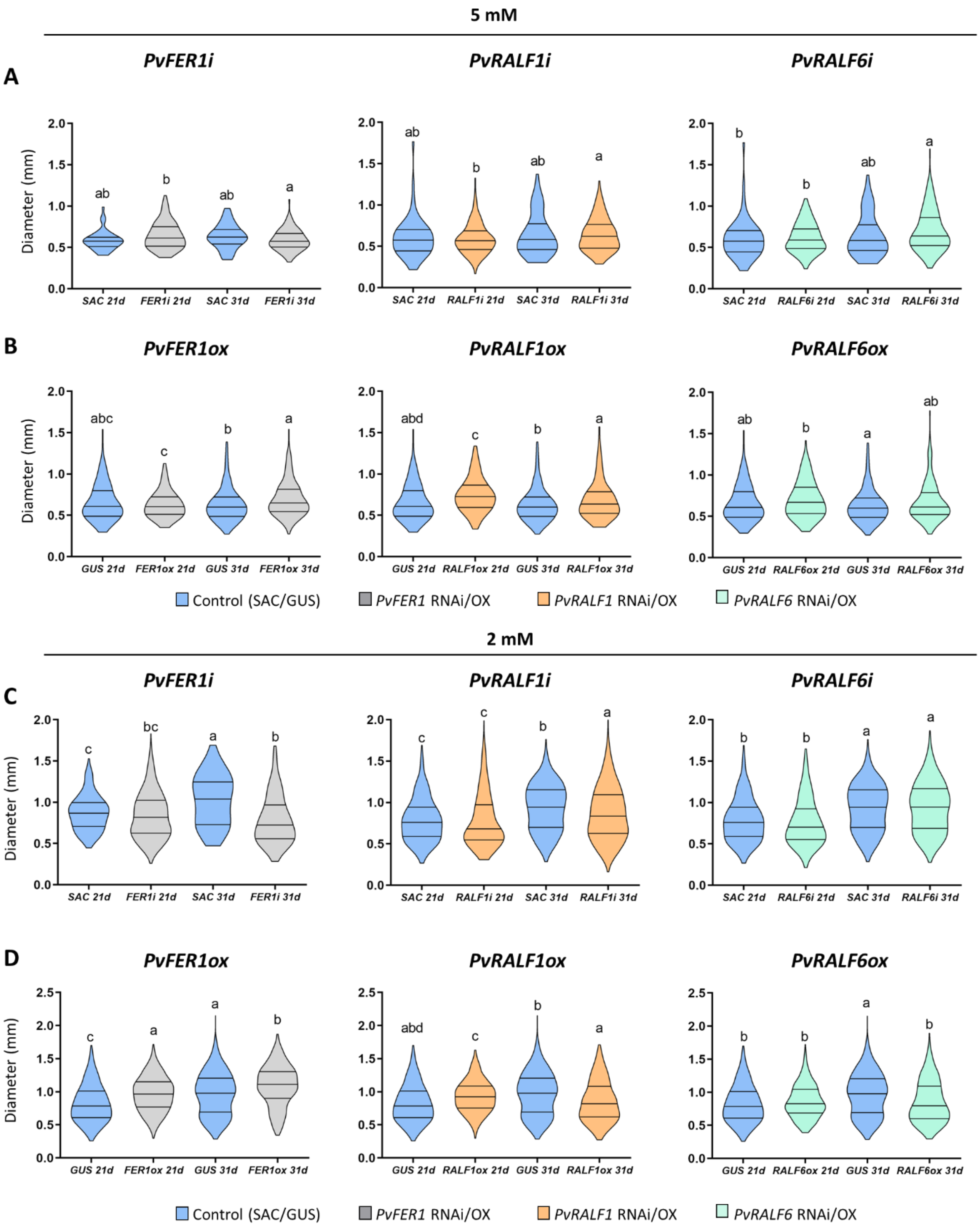

Fig. S12. Analysis of the diameters of nodules generated in roots with silenced and overexpressed *PvFER1*, *PvRALF1* and *PvRALF6* genes under 2 and 5 mM nitrate. Analysis of nodule diameters collected from transgenic roots with silenced (A and C) or overexpressed (B and D) *PvFER1*, *PvRALF1*, or *PvRALF6* and the respective control roots under 5 (A and B) or 2 mM nitrate (C and D) at 21 and 31 dpi. Violin plots represent the distribution of all data ($n \geq 100$), the horizontal line within the box represents the median, and the upper and lower lines delimit the interquartile range. A non-parametric Kruskal-Wallis test

followed by Dunn's multiple comparisons was used to assess significant differences with a P value < 0.05 represented by different letters above the different genotypes.

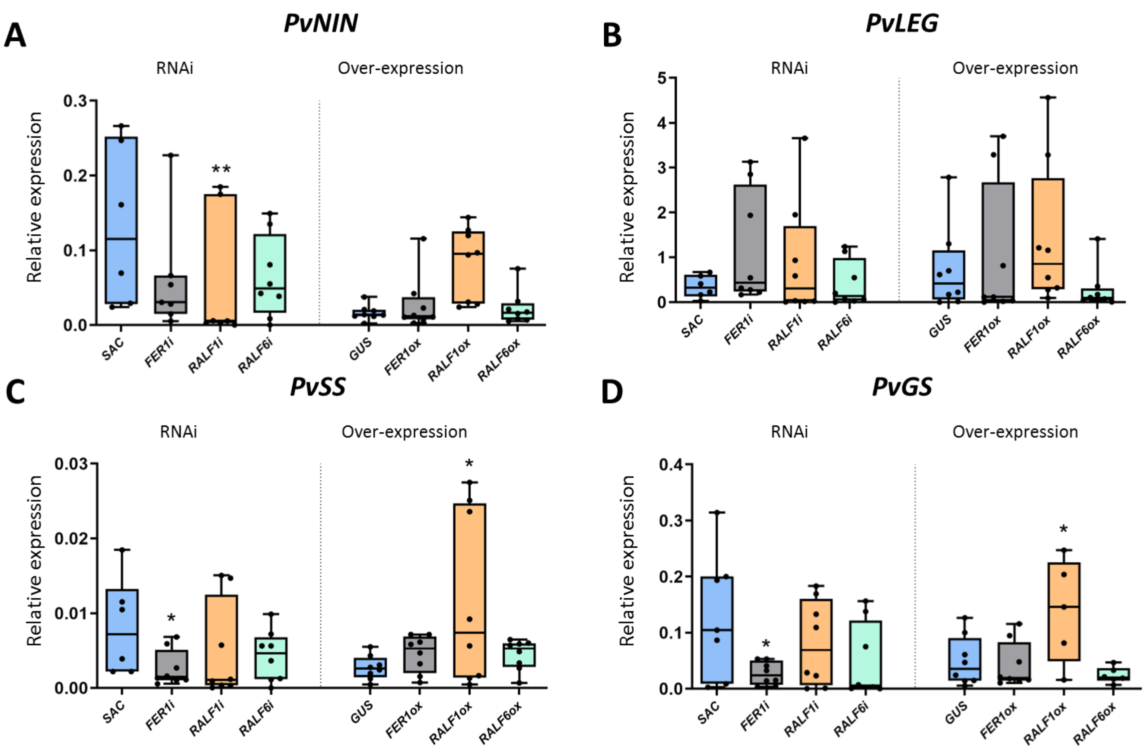

Fig. S13. Accumulation of transcripts levels of the marker genes for nodule organogenesis and metabolism in transgenic bean roots under 5 mM nitrate. The relative expression level of *PvNIN* (A), *PvRLEG* (B), *PvSS* (C), and *PvGS* (D) in transgenic roots at 21 dpi under 5 mM nitrate conditions is shown. *PvFER1i*, *PvRALF1i*, and *PvRALF6i* are silenced transgenic roots, and *PvFER1ox*, *PvRALF1ox*, and *PvRALF6ox* are overexpressed roots. The upper and lower edges of the boxes delimit the first to third quartiles, the horizontal line within the box represents the median, and the whiskers indicate the smallest and largest outlier in the data set. The points represent all data obtained ($n \geq 8$). A non-parametric Mann-Whitney test was used to assess significant differences. *$P < 0.05$, **$P < 0.01$. IDE was used as the reference gene.

Table S1. List of oligonucleotides used for qPCR analysis and vector construction

	Oligonucleotide ID	Oligonucleotide sequence	Amplicon size, bp
**qPCR analysis**	qPCR_FER1_F	TTTGCTGCGTCCCATCGT	137
	qPCR_FER1_R	GCATAACTTCCTGTTGTGTTGGTC	
	qPCR_RALF1_F	CACCATGGCGAAGTCGTGTTCCTT	127
	qPCR_RALF1_R	GGAACCCCATCCCATTCCCAG	
	qPCR_RALF6_F	CACCATGATGTCCCAACCCAGA	127
	qPCR_RALF6_R	ATTGTGTTTCAACAGATTGAGGTCC	
	EF1α_LW	GCACCCAGGCATACTTGAATGACC	146
	EF1α_UP	GGTCATTGGTCATGTCGACTCTGG	
	IDE_LW	GCAACCAACCTTTCATCAGC	156
	IDE_UP	AGAAATGCCTCAACCCTTTG	
	PvNIN_F	GGGGATTCAGAGATTTGCAG	101
	PvNIN_R	AACCCACTCTTGAGCATCGT	
	PvLEG_F	TGGCTGATGCTGCACTTGG	166
	PvLEG_R	GCTGCCAATTCATCGTAGG	
	PvSS_F	CGTCTCTCTTTGTGTTTGGC	154
	PvSS_R	GGATGATGTGCCATCTTCAG	
	PvGS_F	CAGGAGGGGTAACAATAT	189
	PvGS_R	CATCCAACAGGCCACTGAA	
	PvRIC2_F	CCACTAGCCATCTCAAGCTT	111
	PvRIC2_R	CAAGCATGGAGAGTTGGGTT	
	PvTML_F	AGGAGAGTGAAAATGGCAAGG	89
	PvTML_R	GGGAAAAGATAGCACTTGAGGTT	
	PvNARK_F	TTGGTCATCCACATTCACCTC	104
	PvNARK_R	CAGATTTGTTTTGCTGCTCCA	
	PvNIC_F	TTGGGGACAATGGCCAAC	126
	PvNIC_R	ACCTTCGAGTTGGGTCTTC	
	PvCLE35_F	ATCCTTTTCAATGGCAAGTTTG	100
	PvCLE35_R	TGGAAGTGAAGAAGGAAGAGG	
**Promoter analysis**	promFER1_F	CACCTATTTGCTACTAGAAATTGAGT	2004
	promFER1_R	CACGACACAGAATTCCAAACC	
	promRALF1_F	CACCGTAAAAACATATCCTTGACAC	2044
	promRALF1_R	TGATCAGAGGCTTCTCTTTT	
	promRALF6_F	CACCAGGGTTCAAATGAAGATGG	2023
	promRALF6_R	TGTGTGTCGAGAGATGTTTTG	
	FER1ox_F	CACCATGTGGAGCATGAATCG	2701

	FER1ox_R	ACGCCCTTTTGGATTCATGATC	
**Overexpression analysis**	RALF1ox_F	CACCATGGCGAAGTCGTGTTCCTT	352
	RALF1ox_R	GCTCCTGCACCTCGTAATGG	
	RALF6ox_F	CACCATGATGTCCCAACCCAGA	388
	RALF6ox_R	AGTCTTGAAGTCTTGACCTCT	
**RNAi-silencing analysis**	FER1RNAi_F	CACCATCCTCACCTTTATTAGA	123
	FER1RNAi_R	AATTACAATCACGCACACCCA	
	RALF1RNAi_F	CACCATGATGAAACGAGAGATTTG	278
	RALF1RNAi_R	TGATCAGAGGCTTCTCTTTT	
	RALF6RNAi_F	CACCTGACAGTAATGATGGCAC	171
	RALF6RNAi_R	TGTGTGTCGAGAGATGTTTTG	

* The four bases necessary for site-oriented cloning in the pENTR-dTOPO vector are indicated in red.

Table S2. Changes in the expression of *NARK-like*, *CLE-like,* and *TML-like* genes in the *A. thaliana fer1* and *fer4* loss-of-function mutants and in a RALF23-overexpressing line.

ID	*fer1*[¥]	*fer4*[γ]	RALF23 OX[€]
**NARK-LIKE**			
AT1G08590	DOWN	DOWN	DOWN
AT1G09970	UP	UP	UP
AT1G17750	UP	ND	ND
AT1G28440	DOWN	ND	ND
AT1G34110	ND	ND	DOWN
AT1G73080	UP	ND	ND
AT1G75820	DOWN	ND	ND
AT2G33170	UP	ND	ND
AT3G19700	UP	ND	ND
AT3G24240	ND	ND	ND
AT3G49670	DOWN	DOWN	ND
AT4G20140	DOWN	ND	ND
AT4G20270	DOWN	ND	ND
AT4G26540	DOWN	ND	DOWN
AT4G28490	UP	UP	ND
AT5G25930	UP	UP	ND
AT5G48940	ND	ND	ND
AT5G49660	DOWN	ND	ND
AT5G56040	DOWN	ND	ND
AT5G61480	ND	DOWN	ND
AT5G65700	DOWN	ND	UP
AT5G65710	DOWN	ND	ND
**TML-LIKE**			
AT1G14330	UP	ND	ND
AT1G26930	UP	UP	ND
AT1G74510	ND	ND	ND
AT2G02870	UP	ND	ND
AT3G27150	ND	ND	ND
AT5G40680	ND	ND	ND
AT5G60570	ND	DOWN	ND
**CLE-LIKE**			
AT1G06225	ND	ND	ND
AT1G73165	ND	ND	ND
AT2G31081	ND	ND	ND
AT2G31082	ND	ND	ND
AT2G31083	ND	ND	ND
AT2G31085	ND	ND	ND
AT4G18510	ND	ND	ND

* Blue and gray cells indicate down-regulation and up-regulation, respectively. ND, not detected; DOWN, down-regulated; UP, up-regulated. **¥** (Guo et al., 2018); **γ** (Wang et al., 2022); **€** (Tang et al., 2022).
